# Systematic review and meta-analysis on the impact on outcomes of device algorithms for minimizing right ventricular pacing

**DOI:** 10.1093/europace/euae212

**Published:** 2024-08-09

**Authors:** Davide Antonio Mei, Jacopo Francesco Imberti, Marco Vitolo, Niccolò Bonini, Kevin Serafini, Marta Mantovani, Enrico Tartaglia, Chiara Birtolo, Marco Zuin, Matteo Bertini, Giuseppe Boriani

**Affiliations:** Cardiology Division, Department of Biomedical, Metabolic and Neural Sciences, University of Modena and Reggio Emilia, Policlinico di Modena, Via del Pozzo 71, Modena 41121, Italy; Clinical and Experimental Medicine PhD Program, University of Modena and Reggio Emilia, Modena, Italy; Cardiology Division, Department of Biomedical, Metabolic and Neural Sciences, University of Modena and Reggio Emilia, Policlinico di Modena, Via del Pozzo 71, Modena 41121, Italy; Clinical and Experimental Medicine PhD Program, University of Modena and Reggio Emilia, Modena, Italy; Cardiology Division, Department of Biomedical, Metabolic and Neural Sciences, University of Modena and Reggio Emilia, Policlinico di Modena, Via del Pozzo 71, Modena 41121, Italy; Clinical and Experimental Medicine PhD Program, University of Modena and Reggio Emilia, Modena, Italy; Cardiology Division, Department of Biomedical, Metabolic and Neural Sciences, University of Modena and Reggio Emilia, Policlinico di Modena, Via del Pozzo 71, Modena 41121, Italy; Clinical and Experimental Medicine PhD Program, University of Modena and Reggio Emilia, Modena, Italy; Cardiology Division, Department of Biomedical, Metabolic and Neural Sciences, University of Modena and Reggio Emilia, Policlinico di Modena, Via del Pozzo 71, Modena 41121, Italy; Cardiology Division, Department of Biomedical, Metabolic and Neural Sciences, University of Modena and Reggio Emilia, Policlinico di Modena, Via del Pozzo 71, Modena 41121, Italy; Cardiology Division, Department of Biomedical, Metabolic and Neural Sciences, University of Modena and Reggio Emilia, Policlinico di Modena, Via del Pozzo 71, Modena 41121, Italy; Cardiology Division, Department of Biomedical, Metabolic and Neural Sciences, University of Modena and Reggio Emilia, Policlinico di Modena, Via del Pozzo 71, Modena 41121, Italy; Cardiology Unit, Department of Translational Medicine Sant’Anna University Hospital, University of Ferrara, Ferrara, Italy; Cardiology Unit, Department of Translational Medicine Sant’Anna University Hospital, University of Ferrara, Ferrara, Italy; Cardiology Division, Department of Biomedical, Metabolic and Neural Sciences, University of Modena and Reggio Emilia, Policlinico di Modena, Via del Pozzo 71, Modena 41121, Italy

**Keywords:** Algorithms, Atrial fibrillation, Atrioventricular block, Heart failure, Meta-analysis, Pacemaker, Sinus node dysfunction

## Abstract

**Aims:**

Physiological activation of the heart using algorithms to minimize right ventricular pacing (RVPm) may be an effective strategy to reduce adverse events in patients requiring anti-bradycardia therapies. This systematic review and meta-analysis aimed to evaluate current evidence on clinical outcomes for patients treated with RVPm algorithms compared to dual-chamber pacing (DDD).

**Methods and results:**

We conducted a systematic search of the PubMed database. The predefined endpoints were the occurrence of persistent/permanent atrial fibrillation (PerAF), cardiovascular (CV) hospitalization, all-cause death, and adverse symptoms. We also aimed to explore the differential effects of algorithms in studies enrolling a high percentage of atrioventricular block (AVB) patients. Eight studies (7229 patients) were included in the analysis. Compared to DDD pacing, patients using RVPm algorithms showed a lower risk of PerAF [odds ratio (OR) 0.74, 95% confidence interval (CI) 0.57–0.97] and CV hospitalization (OR 0.77, 95% CI 0.61–0.97). No significant difference was found for all-cause death (OR 1.01, 95% CI 0.78–1.30) or adverse symptoms (OR 1.03, 95% CI 0.81–1.29). No significant interaction was found between the use of the RVPm strategy and studies enrolling a high percentage of AVB patients. The pooled mean RVP percentage for RVPm algorithms was 7.96% (95% CI 3.13–20.25), as compared with 45.11% (95% CI 26.64–76.38) of DDD pacing.

**Conclusion:**

Algorithms for RVPm may be effective in reducing the risk of PerAF and CV hospitalization in patients requiring anti-bradycardia therapies, without an increased risk of adverse symptoms. These results are also consistent for studies enrolling a high percentage of AVB patients.

## Graphical Abstract

**Graphical Abstract:**
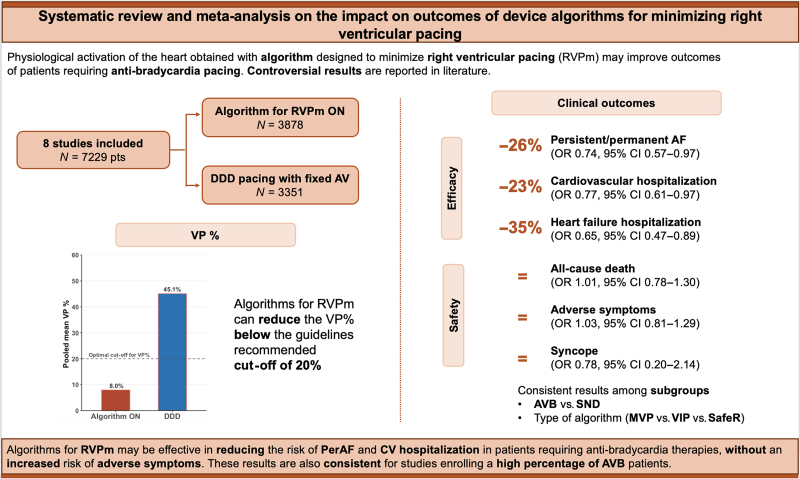

## Introduction

Right ventricular pacing (RVP) is considered and recommended by many clinical guidelines as the treatment of choice for conditions that require anti-bradycardia therapies, including atrioventricular blocks (AVBs) and sinus node disease (SND).^1,2^ In recent years, the use of pacemakers (PMs) has significantly increased.^3,4^ This increase is due to the aging population, improved survival rates among patients with heart conditions, and a reduced rate of complications after implantation.^5–8^ However, various clinical trials have shown that a high percentage of RVP is associated with adverse outcomes, including an increased risk of heart failure (HF) and pacing-induced cardiomyopathy.^9–11^ An RVP percentage >40% has been identified as a significant risk factor for developing these conditions, although clinical guidelines suggest that even a cut-off of 20% may be considered.^1,12,13^

The preservation of the physiological intrinsic ventricular activation is fundamental to prevent the development of pacing-induced cardiomyopathy, thus preventing the occurrence of adverse events. This need has led to the development of strategies for RVP minimization (RVPm).^14^ In recent years, many manufacturers have developed various algorithms to reduce unnecessary RV pacing. These algorithms are primarily based on two mechanisms: (i) progressive prolongation of the AV pacing delay [AV hysteresis (AVH)] and (ii) mode switch modalities that provide AAI pacing with ventricular monitoring and an automatic switch from AAI to standard dual-chamber pacing (DDD) during episodes of AVB. Both mechanisms include DDD backup when needed. Clinical trials have shown that both AVH and AAI-DDD modes can reduce ventricular pacing.^14^ As a result, clinical guidelines recommend reducing unnecessary ventricular pacing using these algorithms for treating patients with conduction disorders.^1,2^ However, data regarding their impact on clinical outcomes are inconsistent. Furthermore, most studies have been conducted in cohorts of patients primarily diagnosed with SND, thus limiting the extent of evidence for AVB patients.^15^ Therefore, we performed a systematic review and meta-analysis to assess the effect of algorithms for RVPm on clinical outcomes and to evaluate their efficacy in SND and AVB populations.

## Methods

The present systematic review and meta-analysis was conducted in accordance with the Preferred Reporting Items for Systematic Reviews and Meta-Analysis (PRISMA) 2020 guidelines.^16^ Details regarding the search strategy, inclusion and exclusion criteria, and the processes of study selection and data extraction are provided in the Supplementary material.

### Quality assessment

Two independent authors (D.A.M. and J.F.I.) performed quality assessment using the Newcastle-Ottawa Scale (NOS) for non-randomized clinical trials and V.2 of the Cochrane ‘Risk of Bias’ (RoB2) tool for randomized controlled trials.^17^ The robvis internet-based graphic generating platform was used to create the risk of bias (ROB) plot with the results from RoB2.^18^ Studies with a NOS ≤7 of the NOS were categorized at significant ROB.^19^

### Definition of outcomes

For our analysis, we considered as relevant endpoint:

1. The impact of RVPm strategies on adverse outcomes compared with DDD patients. Relevant and consistent outcomes included:
  - Persistent/permanent atrial fibrillation (PerAF) was defined as reported by single studies.
  - Cardiovascular (CV) hospitalizations, defined according to a composite of different causes of hospitalization as reported by the studies included in the analysis: HF, tachyarrhythmias, cardioversion, coronary artery disease, pulmonary embolism, stroke, or other CV event.
  - Heart failure hospitalization, reported as a single endpoint where available.
  - All-cause death, defined as death from cardiac or non-cardiac causes.
  - Adverse symptoms, defined as a composite of one between palpitation, dizziness, dyspnoea, fatigue, syncope, and angina.
  - Syncope, where reported as a single endpoint, that was chosen because it represents a more homogeneous and clinically significant adverse event.
2. The percentages of ventricular pacing in the RVPm and DDD groups.

### Statistical analysis

For direct comparison of outcomes, we utilized the Mantel–Haenszel random-effects model to determine pooled estimates reported as odds ratios (ORs) with 95% confidence intervals (CIs). Heterogeneity was assessed using the inconsistency index (*I*^2^), categorized as low (<25%), moderate (25–75%), or high (>75%) based on pre-specified cut-offs.^20^

Sensitivity analyses were conducted using a ‘leave-one-out’ approach, where each study was sequentially removed to assess its impact on pooled estimates and heterogeneity. As per our pre-specified methods (see Supplementary material), studies with more than two groups were consolidated, with DDD used as the control and groups with activated algorithms merged. To strengthen the consistency of the analysis, we also performed a sensitivity analysis including only the group of patients treated with the algorithm considered to be more similar to those used in the other studies included in the present analysis.

To account for potential residual sources of heterogeneity, we also performed several subgroup analyses, according to the type of algorithm for RVPm used, the percentage of patients with AVB (≥ vs. <30% of patients enrolled in the study), and the percentage of female patients included in the studies (≥ vs. <50% of female patients enrolled). We calculated the *P*-value for interaction (*P*_int_) to test whether the effect of the treatment differed significantly between subgroups. The *P*_int_ helps in identifying potential differential effects across various subgroups, providing insights into possible sources of heterogeneity.

The means of RVP percentages were pooled using the random-effects model with inverse variance weighting. For studies reporting median and interquartile range, we applied the method described by Wan *et al.*^21^ to estimate mean and standard deviation.

Publication bias was through visual inspection of funnel plots. All the statistical analyses were performed using R 4.2.2 for MacOS (The R. Foundation, 2020), using ‘dmetar’ package.

## Results

### Summary of the studies

**Figure 1:**
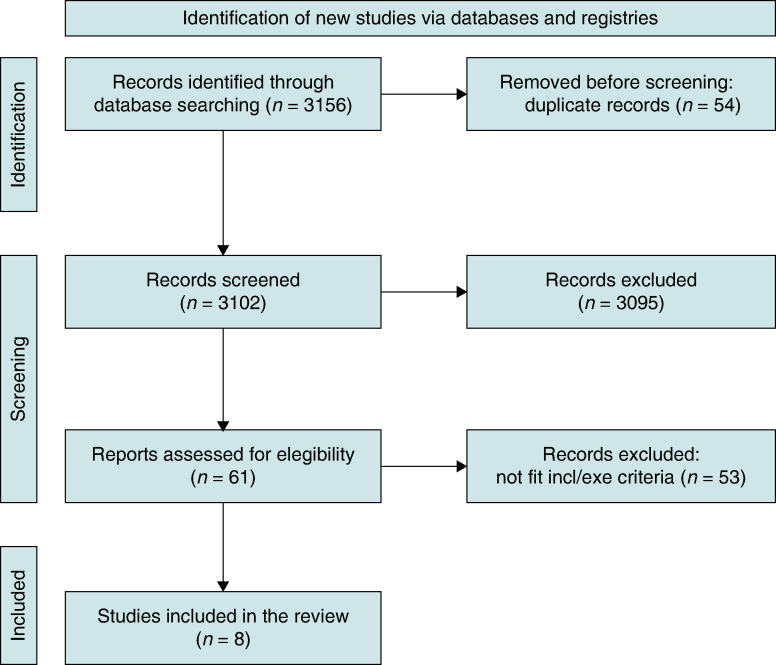
PRISMA flowchart of the study.

The literature search initially identified 3156 studies. After removing duplicates and screening titles and abstracts, 61 full texts were assessed for eligibility, resulting in 8 studies included for quantitative synthesis (*Table 1* and *Figure 1*).^22–29^ Of these, seven studies^22–28^ were randomized trials, while one study^29^ was based on observational multicentre data. The duration of follow-up in the studies ranged from 12 to 36 months (*Table 1*). Managed Ventricular Pacing (MVP) and SafeR algorithms were used in three studies, respectively, while Ventricular Intrinsic Preference (VIP) algorithm was used in two. Mechanisms of function, along with potential advantages and disadvantages of these algorithms, are detailed in Supplementary material online, *Table S1*.

**Table 1:** Baseline characteristics of studies and patients

Study, year	Study type	Inclusion/criteria	Exclusion	Algorithm	*N*	Age^a^	Female, *N* (%)	AVB, *N* (%)	SND, *N* (%)	LVEF (%)	FU months	Primary endpoint	Secondary endpoint
Sweeney *et al.*, 2007^22^	RCT	SND	Any AVB and persistent AF and AVB	Search AV or MVP	RVPm, 530	72.1 (11.9)	264 (50)	0 (0)	530 (100)	58 (9.5)	20	PerAF	HFH and %VP
					DDD, 535	72.6 (11.5)	281 (53)	0 (0)	535 (100)	58 (10)			
Davy *et al.*, 2012^23^	RCT	SND/parox AVB	Permanent AVB and PerAF	SafeR	RVPm, 287	73.1	146 (51)	86 (30)	128 (48)	NR	12	%VP	PerAF
					DDD, 135	73.2	69 (51)	42 (31)	62 (46)	NR			
Boriani *et al.*, 2014^24^	RCT	SND/parox AVB	III AVB and permanent AF	MVP	RVPm, 766	74 (9)	398 (52.0)	74 (9.7)	648 (84.6)	57 (21)	24	Death + PerAF + CV hosp	Death, PerAF, CV hosp
					DDD, 385	73 (9)	180 (46.7)	39 (10.1)	118 (30.6)	56 (10)			
Botto *et al.*, 2014^25^	RCT	Replacement of DDD device with %VP > 40%	III AVB and permanent AF	MVP	RVPm, 299	75 (11)	125 (41.8)	71 (23.7)	183 (61.2)	54 (12)	24	CV hosp	Death, PerAF
					DDD, 306	73 (9)	115 (35.6)	68 (22.2)	186 (60.8)	52 (13)			
Bauer *et al.*, 2015^26^	RCT	SND/parox AVB	Permanent AVB and PerAF	VIP	RVPm, 195	NR	NR	NR	NR	NR	12	%VP	Death, PerAF
					DDD, 194	NR	NR	NR	NR	NR			
Stockburger *et al.*, 2015^27^	RCT	SND/parox AVB	Permanent AVB and permanent AF	SafeR	RVPm, 314	71.8 (12.2)	132 (42.0)	145 (46.2)	167 (53.2)	59 (9)	36	%VP and HFH + PerAF	HFH, PerAF
					DDD, 318	72.9 (9.8)	149 (46.8)	157 (49.4)	160 (50.3)	58 (8)			
Thibault *et al.*, 2015^28^	RCT	SND/parox AVB	Permanent AF and AVB	SafeR	RVPm, 191	70.0 (10)	67 (35.1)	78 (40.8)	136 (71.2)	61 (9)	36	PerAF	CV hosp
					DDD, 182	72.0 (11)	73 (40.1)	75 (41.2)	137 (75.3)	60 (10)			
Arnold *et al.*, 2023^29^	Obs	SND/parox AVB	Permanent AF and AVB	VIP	RVPm, 1296	74.0 (12)	613 (47.3)	530 (40.9)	548 (42.3)	58 (10)	12	%VP and CV death + HFH + PerAF	CV death, HFH, PerAF
					DDD, 1296	76.0 (12)	555 (42.8)	444 (34.3)	629 (48.5)	57 (10)			

AF, atrial fibrillation; AVB, atrioventricular block; CV, cardiovascular; FU, follow-up; HFH, heart failure hospitalization; LVEF, left ventricular ejection fraction; MVP, Managed Ventricular Pacing; NR, not reported; Obs, observational; PerAF, persistent/permanent atrial fibrillation; RCT, randomized clinical trial; RVPm, right ventricular pacing minimization; SND, sinus node disease; VIP, Ventricular Intrinsic Preference; VP, ventricular pacing.

^a^Mean (standard deviation).

Two trials included in our analysis randomized patients into three groups. In the study by Boriani *et al.*,^24^ patients were randomized into: (i) standard DDD (DDD control group); (ii) MVP activated only (MVP group); and (iii) atrial preventive pacing (ATP) with MVP activated (MVP + ATP group). For the purpose of our analysis, as pre-specified in the methods, we pooled the two groups with MVP algorithm active and compared them with the DDD group. As specified in our methods, we pooled the two groups with the MVP algorithm active and compared them with the DDD group. This approach ensured all populations treated with the RVPm algorithm were considered while avoiding double counting of the DDD group. In a pre-specified sensitivity analysis, we included only patients randomized to the MVP group.

In another trial, Davy *et al.*^23^ randomized patients into (i) DDD group with a long AV delay (250 ms after sensed events, 300 ms after paced events); (ii) SafeR group; and (iii) DDD with automatic mode conversion (DDD/AMC group). For our main analysis, we pooled the SafeR and DDD/AMC groups and performed a sensitivity analysis excluding patients randomized to the DDD/AMC group. We excluded from the meta-analysis one study by Chen *et al.*^30^ that randomized patients to MVP vs. Search AV algorithm. This was necessary, in order to be as consistent as possible with other trials that compared patients with RVPm algorithm with DDD with fixed AV. Similarly, we excluded an analysis by Pastore *et al.*^31^ because the group of control was DDD pacing implanted in the His bundle.

### Baseline characteristic

In total, 7229 patients were included in the meta-analysis. Right ventricular pacing minimization algorithms were employed in 3878 patients (VIP in 1491, MVP in 1595, and SafeR in 792). *Table 1* reports the baseline characteristics of the different studies and the main characteristics of patients enrolled. In four studies,^23,27–29^ more than one-third of patients enrolled were implanted because of intermittent/paroxysmal AVB. Permanent AVB was uniformly excluded as a criterion across all studies. The study by Botto *et al.*^25^ uniquely included patients referred for generator replacement, as well as those with implanted implantable cardioverter-defibrillators (ICDs).

### Outcomes of interest

#### Persistent/permanent atrial fibrillation

**Figure 2:**
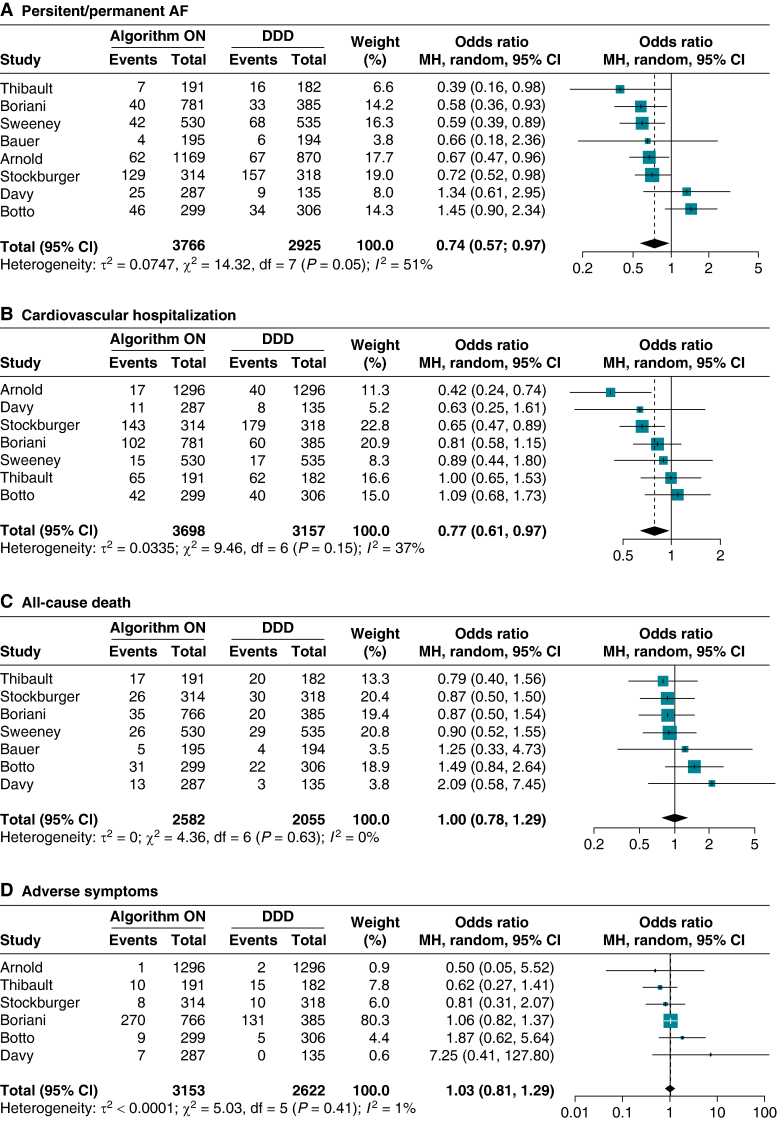
Effect of algorithms for RVPm vs. standard DDD pacing on adverse outcomes in patient requiring anti-bradycardia therapies. (*A*) Persistent/permanent AF. (*B*) Cardiovascular hospitalization. (*C*) All-cause death. (*D*) Adverse symptoms. AF, atrial fibrillation; CI, confidence interval; DDD, dual-chamber pacing; MH, Mantel–Haenszel; RVPm, right ventricular pacing minimization.

All eight studies included in our analysis reported data on the development of PerAF.^22–29^ Patients treated with RVPm algorithms demonstrated a reduced likelihood of PerAF occurrence (OR: 0.74, 95% CI 0.57–0.97, *Figure 2A*), with moderate heterogeneity observed (*I*^2^: 51%). Sensitivity analysis using the leave-one-out approach indicated minimal influence of individual studies on the overall pooled estimates (see Supplementary material online, *Figure S1A*). Conversely, exclusion of the study by Botto *et al.*^25^ significantly reduced heterogeneity, consistently showing lower PerAF risk for RVPm-treated patients (OR: 0.66, 95% CI 0.56–0.79; *I*^2^: 0%, Supplementary material online, *Figure S1A*). Similarly, sensitivity analyses excluding the third arms of studies by Boriani *et al.*^24^ and Davy *et al.*^23^ yielded comparable pooled estimates to the main analysis (see Supplementary material online, *Figure S2A*).

**Figure 3:**
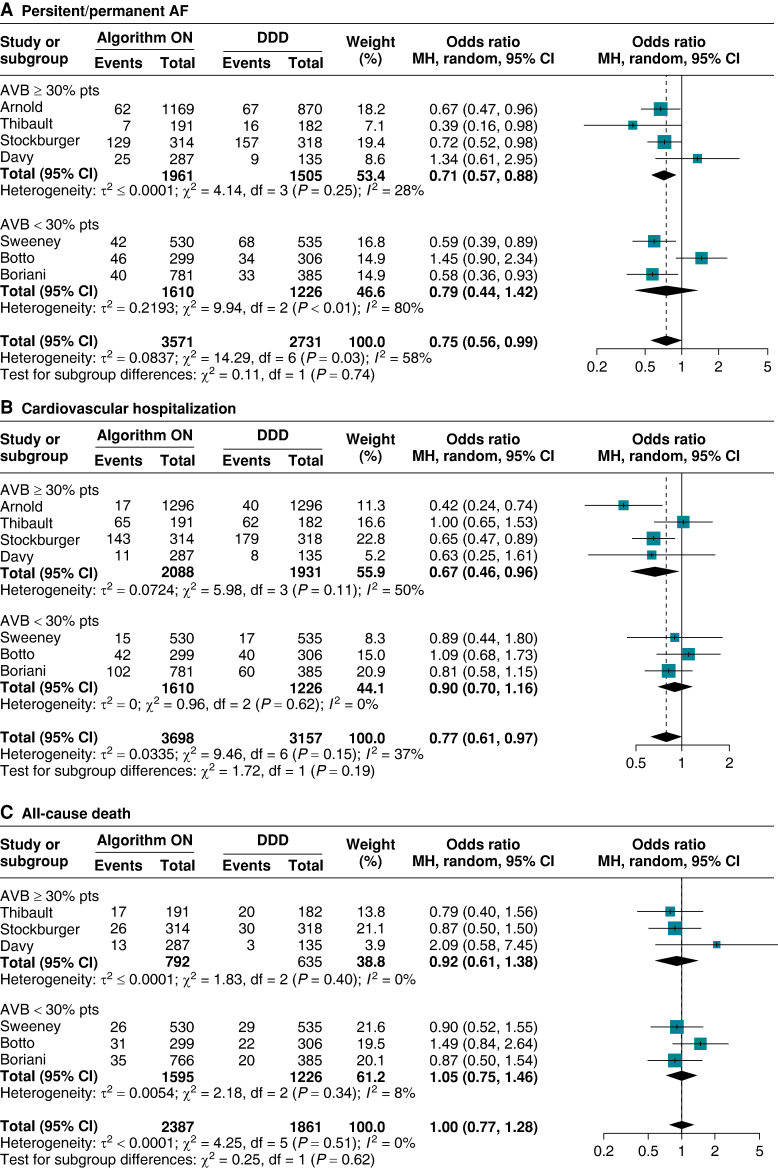

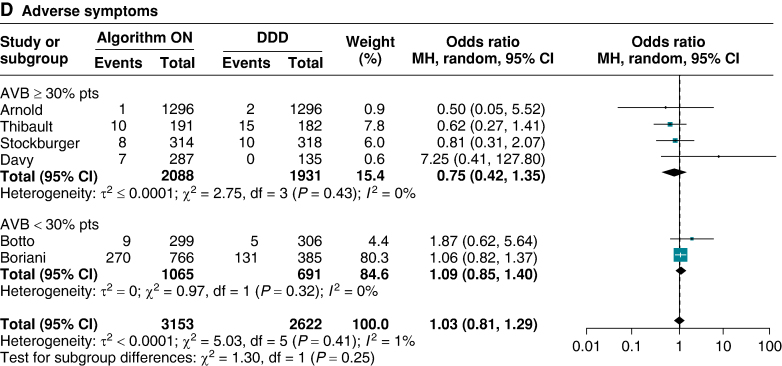
Subgroup analysis for outcomes according to the proportion of patients with AVB in the original cohorts. (*A*) Persistent/permanent AF. (*B*) Cardiovascular hospitalization. (*C*) All-cause death. (*D*) Adverse symptoms. AF, atrial fibrillation; AVB, atrioventricular block; CI, confidence interval; DDD, dual-chamber pacing; MH, Mantel–Haenszel.

Subgroup analysis revealed no significant interaction effect between the type of algorithm used and PerAF risk (*P*_int_ = 0.88, Supplementary material online, *Figure S3A*). However, lower heterogeneity was observed among studies employing the VIP algorithm compared to MVP and SafeR algorithms (VIP *I*^2^: 0%; MVP *I*^2^: 80%; SafeR *I*^2^: 50%). Similarly, no significant differences were found based on the proportion of patients with AV block (*P*_int_ = 0.74, *Figure 3A*) or the proportion of female patients included (*P*_int_ = 0.64, Supplementary material online, *Figure S4A*).

#### Cardiovascular and heart failure hospitalization

Seven studies reported data on the occurrence of CV hospitalization.^22–25,27–29^ Patients treated with algorithms for RVPm demonstrated reduced odds of CV hospitalization with moderate heterogeneity (OR 0.77, 95% CI 0.61–0.97, *I*^2^: 37% *Figure 2B*). Sensitivity analysis with the ‘leave-one-out’ approach showed no significant influence of a single study on the pooled estimates; however, the study by Arnold *et al.*^29^ importantly contributed to overall heterogeneity (see Supplementary material online, *Figure S1B*). Consistent results were observed in the second sensitivity analysis (OR 0.74, 95% CI 0.58–0.96, *I*^2^: 47%, Supplementary material online, *Figure S2B*).

Subgroup analysis did not reveal a significant interaction between different types of algorithms used (*P*_int_ = 0.06, Supplementary material online, *Figure S3B*) nor according to the proportion of patients with AV block or female participants in the original trials (*P*_int_ = 0.19, *Figure 3B*, *P*_int_ = 0.77, Supplementary material online, *Figure S4B*, respectively).

To further explore the association between RVPm strategies and adverse events, we specifically assessed hospitalization due to HF. Overall, five studies reported about HF hospitalization.^22,23,27–29^ Consistent with the main analysis on CV hospitalization, the use of RVPm algorithms was associated with a lower risk of HF hospitalization (OR: 0.65, 95% CI 0.47–0.89, *I*^2^: 47%, Supplementary material online, *Figure S5A*).

#### All-cause death

Seven studies included in the meta-analysis reported on the occurrence of all-cause death.^22–28^ The use of RVPm algorithms did not result in a significant effect on the risk of all-cause death with low heterogeneity (OR: 1.01, 95% CI 0.78–1.30, *I*^2^: 0%, *Figure 2C*). Consistent results have been found at the two sensitivity analyses (see Supplementary material online, *Figures S1C* and *S2C*). No significant interaction effect has been found at subgroup analysis for the type of algorithm used and for the percentage of AVB or female patients (see Supplementary material online, *Figure S3C*, and *Figure 3C* and Supplementary material online, *Figure S4C*).

#### Adverse symptoms and syncope

Information on adverse symptoms was available from six studies^23–25,27–29^: RVPm did not increase the risk of experiencing adverse symptoms (OR:1.03, 95% CI 0.81–1.29, *I*^2^ = 1%, *Figure 2D*). These findings were consistent in sensitivity analyses using the leave-one-out approach, with no single study significantly affecting the pooled effects or heterogeneity (see Supplementary material online, *Figure S1D*). Similar results were also found at the second sensitivity analysis (see Supplementary material online, *Figure S2D*). Subgroup analyses did not reveal significant differences across the categories assessed (see Supplementary material online, *Figure S3D*, and *Figure 3D* and Supplementary material online, *Figure S4D*).

To further investigate the effect of RVPm on adverse symptoms, we specifically analysed syncope events reported in five studies^23,24,27–29^: the use of algorithms was not associated with an increased risk of syncope (OR: 0.78, 95% CI 0.20–2.14, *I*^2^ 47%, Supplementary material online, *Figure S5B*).

#### Right ventricular pacing percentage

**Figure 4:**
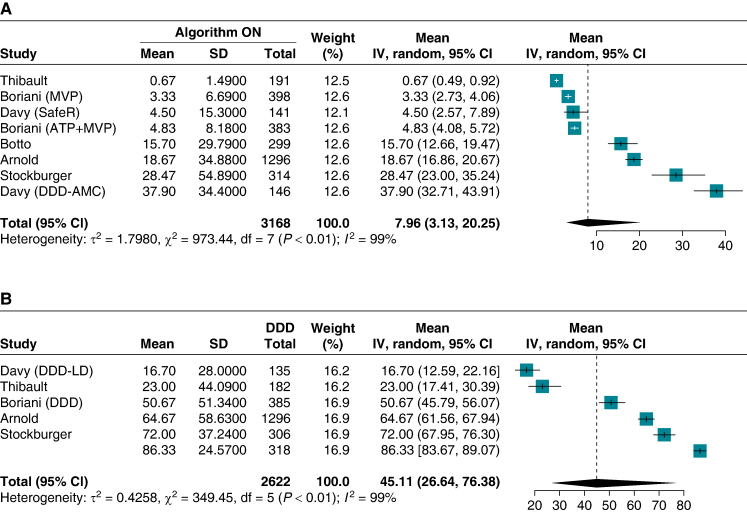
Pooled mean of right ventricular pacing. (*A*) Algorithm for RVPm ON. (*B*) The DDD group. ATP+MVP, atrial preventive pacing with MVP activated; CI, confidence interval; DDD-AMC, DDD with automatic mode conversion; DDD-LD, DDD group with a long AV delay; IV, inverse variance; SD, standard deviation.

In total, six studies provided data on the percentage of RVP. The pooled mean RVP percentage for patients with RVPm algorithms activated was 7.96% (95% CI 3.13–20.25, *Figure 4A*). In contrast, patients in the DDD pacing mode had a pooled mean RVP percentage of 45.11% (95% CI 26.64–76.38, *Figure 4B*). Two studies^23,28^ showed only a modest reduction in the overall DDD pacing percentage. In both cases, the DDD control group was programmed with a long AV delay, thus reducing the overall RV pacing burden, albeit with an expected prolongation of the PR interval.

#### Bias assessment

Visual inspection of the funnel plots revealed potential asymmetry for CV hospitalization and adverse symptoms, with missing studies in the bottom right and left of the plot. Similarly for all-cause death, there may be missing studies in the bottom left. We did not observe publication bias for the other outcomes. Funnel plots are reported in Supplementary material online, *Figure S6*.

Quality assessment of the studies is reported in Supplementary material online, *Figure S7*. Of the seven randomized trial, two of them^23,26^ arise concerns regarding the ROB. Similarly, a significant ROB was also present also for the observational study,^29^ mainly because of study design.

## Discussion

The main findings of this systematic review and meta-analysis are as follows: (i) among patients requiring anti-bradycardia therapies, the use of algorithm for RVPm is effective and is associated with a reduce risk of PerAF, CV hospitalization, and HF hospitalization; (ii) this strategy seems to be also safe, without an increased risk of death, adverse symptoms, and syncope; (iii) the efficacy of RVPm algorithms was consistent across patients with SND and AVB, as well as with different algorithm types such as AVH and mode switching; (iv) the algorithms successfully reduced RVP below the recommended threshold of 20%, which is critical for minimizing pacing-induced adverse effects.

Patients with previous AF implanted with DDD devices are known to have a higher risk of progressing to persistent or permanent AF.^32^ Modern cardiac implantable electronic devices enable continuous monitoring of atrial rhythm.^33,34^ which allows for detection of subclinical AF episodes in nearly one-third of patients.^35^ Despite being asymptomatic in most cases, these episodes represent an important, independent predictor of stroke and the development of clinical persistent AF.^36–38^ Recent studies underscore the significance of AF progression, linking it to adverse outcomes with higher AF burden.^39^ In this scenario, a tool that is available and provided in patients implanted with PM and that has been shown to significantly reduce the progression to PerAF can be of clinical utility for the management of patients requiring anti-bradycardia therapies. Our results are in contrast with those reported by a previous meta-analysis by Shurrab *et al.*^40^ that reported a no significant effect of pacing algorithms compared with DDD pacing (OR 0.84, 95% CI 0.57–1.24, *P* = 0.38). In our study, the increased sample size of 7229 patients compared to the sample of 3487 patients in Shurrab *et al.*’s analysis enabled us to reduce the standard error and achieve statistical significance. However, we acknowledge that the CIs in our study remain relatively wide, reflecting the inherent variability among the included studies and the diversity of algorithms tested. Nonetheless, the statistical significance observed in our results suggests a consistent beneficial effect of pacing algorithms.

The physiological mechanism that links RV pacing and AF is yet not completely understood. There is some evidence that electromechanical may play an important role. First, RV pacing has been shown to cause increases in atrial pressure and size, as well as to determine the development of electrophysiological substrate that could facilitate the development of AF.^41–43^ Second, an important role may be played by mitral regurgitation due to papillary muscle desynchronization.^44^

Our analysis also demonstrated a reduction in CV hospitalization for patients treated with algorithms for RVPm. There are several plausible explanations for these findings. First, the outcome CV hospitalization included in many studies the hospitalization for AF cardioversion, hence being strictly linked with the reduction in the overall progression to PerAF. Secondly, we also demonstrated that algorithms significantly reduced the risk of hospitalization for HF. These results align with the hypothesis that a high percentage of RV pacing is associated with an increased risk of pacing-induced cardiomyopathy and episodes of HF.^10,45^ A recent consensus document of the Hearth Rhythm Society^13^ recommends cardiac physiologic pacing [including both cardiac resynchronization therapy (CRT) and conduction system pacing (CPS)] for patients with left ventricular ejection fraction (LVEF) from 36 to 50% (class IIa, level of evidence B) and also for those with normal LVEF (class IIb, level of evidence B). However, both strategies (CRT and CSP) may require longer procedural time and may be not applicable in certain circumstances (e.g. impossibility of coronary sinus cannulation for CRT). Furthermore, we still need data regarding the long-term durability and efficacy of CSP.^46^ In this context, algorithms for RVPm can serve as a valuable alternative that can be activated when deemed appropriate for patients implanted with a standard DDD pacing system.

Importantly, the patients included in our analysis had average LVEF values >50%. Therefore, the beneficial effects of algorithms for RVPm appear to be of value to patients without classical indications for an ICD or CRT devices, according to current guidelines.

However, it is important to consider potential unwanted side effects associated with these strategies. Both AVH and mode switch algorithms can lead to a significant increase in the PR interval. Patients requiring anti-bradycardia therapies often have markedly prolonged AV intervals, sometimes exceeding 300 ms, especially for those with transient AV block. As a matter of fact, a *post hoc* analysis of the DANPACE trial showed a higher long-term incidence of AF for those individuals with a PR interval >180 ms.^47^ Similarly, a *post hoc* analysis of the MINERVA trial showed that the positive effects of RVPm algorithms on AF incidence were observed primarily in patients with a PR interval <180 ms.^48^ Unfortunately, since the individual studies included in our meta-analysis did not report outcomes according to the baseline PR interval, we were unable to conduct a detailed analysis based on this parameter. Nevertheless, considering the available data, it appears crucial a balance between preserving physiological ventricular activation and ensuring an optimal AV interval to better manage patients with implanted devices.

Another consideration is that mode switch algorithms, by allowing the loss of some P-waves (as illustrated in Supplementary material online, *Table S1*), may raise concerns among electrophysiologists regarding potential side effects and the occurrence of syncope. However, an important finding from our analysis is that patients treated with algorithms were not at a higher risk of experiencing adverse symptoms or syncope, indicating that their use is not only effective but also safe. This underscores the overall favourable risk–benefit profile of RVPm algorithms in clinical practice.

Lastly, we exploratory investigated possible differences between different algorithms and between SND and AVB patients. Our subgroup analysis indicated that all algorithms were effective in reducing the risk of PerAF and CV hospitalization. Furthermore, when examining the interaction according to the proportion of patients with AVB, we found a consistent effect of the algorithms on reducing PerAF and CV hospitalization, with similar odds for the probability of adverse symptoms. Albeit no definitive conclusions can be drawn from these results, our analysis further extends the current literature, providing evidence for the safety and efficacy of algorithms also in patient with AVB individuals.^15^ These results are also in line with those reported by Arnold *et al.*^29^ that showed a significant reduction in the incidence of persistent AF also for those AVB patients treated with VIP algorithms (4.5% vs. 1.8%, *P* = 0.023). Consistently, subgroup analysis of the MINERVA trial^24^ did not show significant difference between patient with and without AVB among those treated with MVP algorithm.

Our meta-analysis may have several important clinical implications. We showed that algorithms are an effective strategy to reduce the RVP below the 20%, which is the cut-off suggested in more recent consensus.^13^ Algorithms are already provided in standard DDD PM and can be activated or deactivated as needed based on clinical indications. Hence, the use of a tool that can reduce both progression to PerAF and HF hospitalization may be of great utility in everyday clinical practice, even in the era of physiological pacing. Moreover, reducing the amount of RV pacing can also be beneficial for extending the battery life of PMs, potentially reducing the frequency of device replacements and associated risks, such as infections.^49,50^ Although our analysis did not specifically address battery longevity, we recognize this as an important area for future research. Further studies are required to evaluate the use of these algorithms in association with CSP: the ongoing PhysioVP-AF study will compare CSP vs. RVPm algorithms and will provide further knowledge for the treatments of patients with conduction disorders.

### Study limitations

Our analysis has several limitations that should be acknowledged. Crossover of patients between the groups in randomized trials, upgrade to a different device, and the loss of follow-up may partly affect the results of our study. Even though we performed subgroup analysis, our results cannot be generalized to SND- and AVB-specific populations. One study, by Botto *et al.*, included only patients referred for generator replacement and also included ICD-implanted individuals. These patients were older and had a lower ejection fraction, which significantly differed from the patient populations included in the other studies. This difference may explain why we observed a significant reduction in heterogeneity of the effect upon excluding the Botto *et al.* study in our analysis of PerAF occurrence. We performed several sensitivity analyses with the leave-one-out approach to address this issue, finding consistent pooled estimates. We performed several sensitivity analyses with the leave-one-out approach to address the problem, finding consistent pooled estimates. Even though we tried to homogenize as much as possible the control group, still some heterogeneity across studies in the programming of the standard DDD pacing is still present. Due to limited data, we could not evaluate the impact of population baseline differences and other potential residual confounders on pooled estimates. Some heterogeneity is also present across studies for the CV hospitalization considered. However, the results of our analysis only for HF hospitalization are consistent with the main analysis. Despite our best efforts to include any relevant cohort in our analysis, it is possible that some studies were not included (e.g. because not captured by our search strategy or excluded for irrelevance according to the abstract): in particular, we performed our search only on PubMed. However, this database is the most popular and used one and the probability of not having included relevant study is low. One more limitation is that we could not perform an analysis according to the PR duration at the baseline since many studies did not report the data. Additionally, the composite reporting of adverse symptoms, lacking systematic data on individual symptoms, posed challenges. To mitigate this, we focused on syncope, a more uniformly reported and clinically significant adverse event, in our meta-analysis.

Lastly, the inclusion of one observational study introduces an unavoidable residual bias due to the lack of randomization. However, our leave-one-out sensitivity analysis showed that our results remained consistent even when the observational study was excluded, thereby validating the robustness of our findings. Moreover, other factors not reported in the studies might have impacted the outcomes. For instance, RV lead position and patient comorbidities could significantly influence clinical outcomes but were not systematically accounted for in the included studies.

## Conclusions

In patients with an indication for anti-bradycardia therapies implanted with a dual-chamber device, the use of algorithm for RVPm is associated with a lower risk of developing PerAF, CV hospitalization, and HF hospitalization. The use of algorithms was not associated with an increased risk of adverse symptoms and syncope. The benefit of these strategies may be both for SND and AVB patients.

## Supplementary Material

euae212_Supplementary_Data

## Data Availability

The data underlying this article are available in the article and in its online supplementary material.
