# Supplementary material for: Systematic review and meta-analysis on the impact on outcomes of device algorithms for minimizing right ventricular pacing
Source: Europace. 2024 Aug 9;26(8):euae212. doi: 10.1093/europace/euae212 (PMC11346371; doi:10.1093/europace/euae212)
Supplement: euae212_Supplementary_Data [file euae212_Supplementary_Data.docx]

**Systematic review and meta-analysis on the impact on outcome of device algorithms for minimizing right ventricular pacing**

Davide Antonio Mei^1,2,^ MD, Jacopo Francesco Imberti^1,2^ MD, Marco Vitolo^1,2^ MD,

Niccolò Bonini^1,2^ MD, Marta Mantovani^1^ MD, Enrico Tartaglia^1^ MD,

Kevin Serafini^1^ MD, Chiara Birtolo^1^ MD, Marco Zuin^3^ MD,

Matteo Bertini^3^ MD PhD, Giuseppe Boriani^1^ MD PhD

**Supplementary Methods**

*Search Strategy*

A systematic and comprehensive literature search was performed on MEDLINE (accessed through PubMed) database. The search string used to perform the systematic review was: (Suppression OR minimizing OR reducing OR decreasing OR unnecessary) AND ventricular pacing AND (pacemaker OR implantable cardioverter defibrillator). Regularly updated searches were performed during preparation of the manuscript until 30 March 2024.

*Study Selection*

According to titles and abstracts, four co-authors (MM, KS, ET and CB) systematically and sequentially screened independently all articles retrieved from the literature search. Each article included after the first screening phase was then evaluated according to full-text eligibility. Disagreements were resolved by collegial discussion with a third co-author (DAM). The selection process was performed using a standardized, web-based platform (Rayyan Systems Inc., Cambridge, MA,USA).

*Inclusion and Exclusion Criteria*

According to our primary aim, the main inclusion criteria were: (i) studies that compared algorithms for RVPm to standard dual chamber (DDD), irrespective of study design; ii) included adult population > 18 years old; and iii) provided data on outcomes of interest. We excluded articles not in English, conference abstracts, letters, comments, editorials, case reports, crossover studies, systematic reviews, and meta-analysis. In the case of two or more studies based on the same cohort of patients, we selected the most recently published one that reported data regarding the outcome of interest.

*Data Extraction*

Three co-authors (DAM, MM and KS) independently extracted data from the studies included through a standardized electronic form. We extracted data on sample size, numbers of patients with RVPm algorithm and DDD, type of algorithm used, mean or median age, the proportion of females, the proportion of patients with AVB and SND and mean or median percentage of RV pacing at follow-up. Additionally, we extracted outcome data (persistent/permanent atrial fibrillation, Cardiovascular hospitalization, all-cause death, adverse symptoms and syncope) according to RVPm strategy, when available. For those studies with more than 2 groups, the DDD group was used as control, while the groups with an algorithm for RVPm activated were pooled together: this was done in order to avoid double counting of a single group on the overall pooled estimate.

**SUPPLEMENTARY FIGURES**

**Supplementary Figure 1.** Leave one out analysis for adverse outcomes.

Panel A: persistent/permanent AF; Panel B: cardiovascular hospitalization; Panel C: all-cause death; Panel D: adverse symptoms.


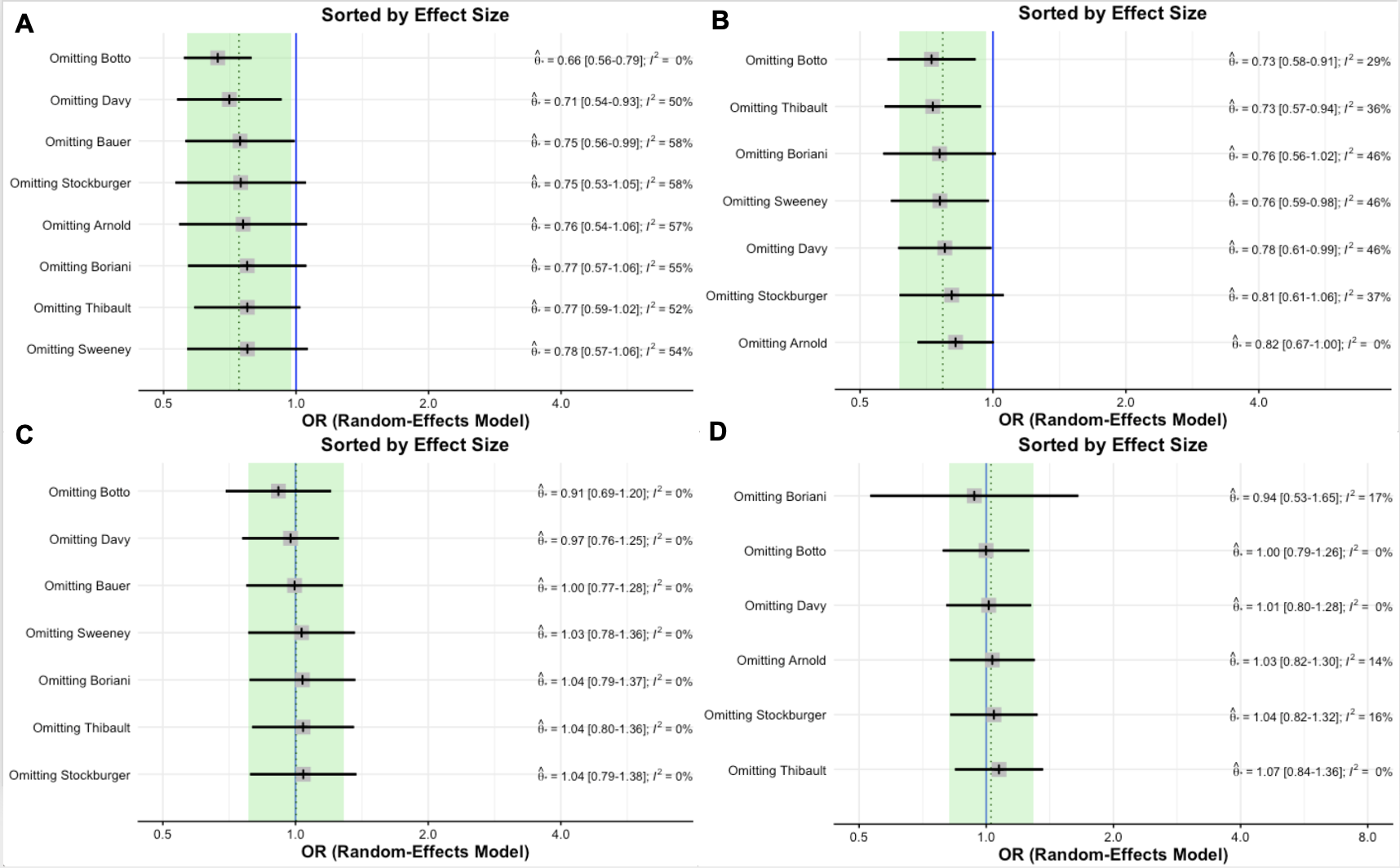


Legend. OR, odds ratio.

**Supplementary Figure 2.** Sensitivity analysis removing the third group for Boriani et al and Davy et al.

Panel A: persistent/permanent AF; Panel B: cardiovascular hospitalization; Panel C: all-cause death; Panel D: adverse symptoms.


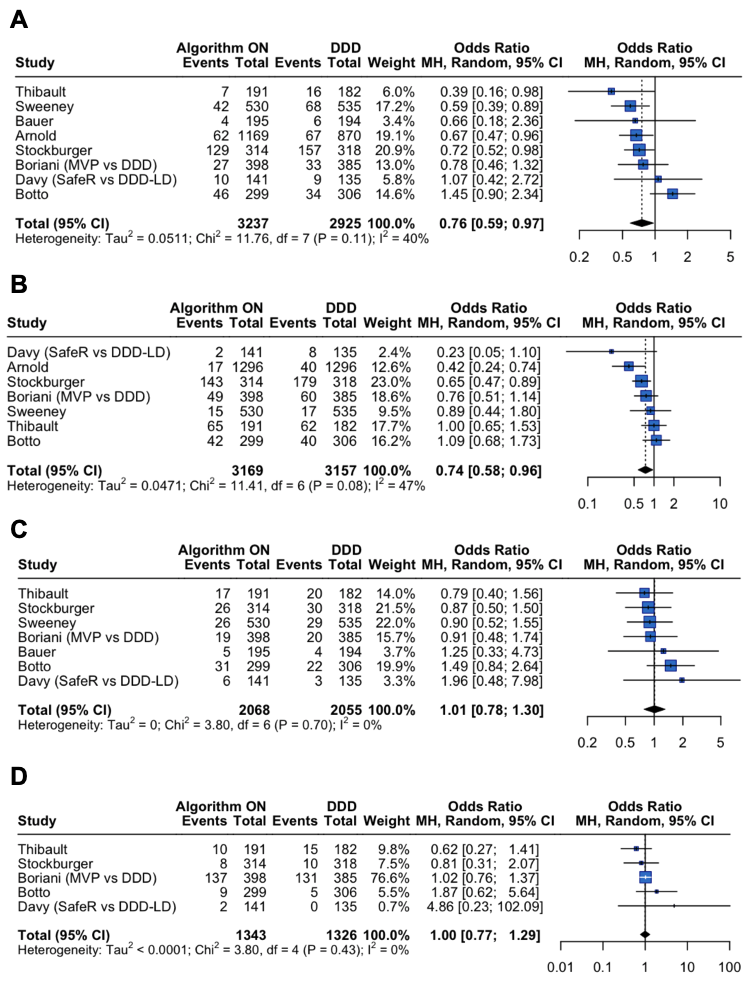


**Legend.** CI, confidence interval; MH, Mantel-Haenszel; RVPm, right ventricular pacing minimization.

**Supplementary Figure 3.** Subgroup analysis for outcomes according to type the algorithm used.

Panel A: persistent/permanent AF; Panel B: cardiovascular hospitalization; Panel C: all-cause death; Panel D: adverse symptoms.


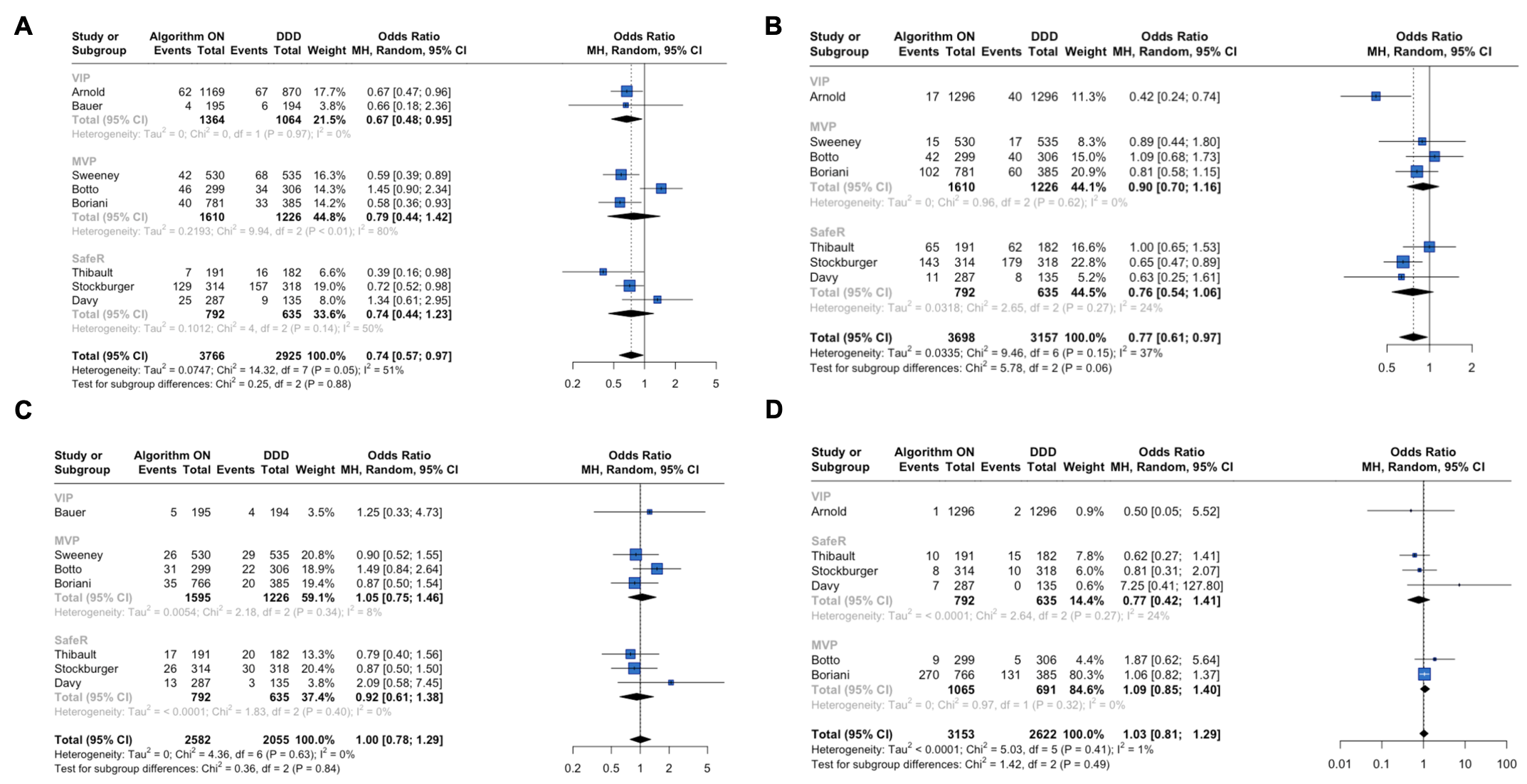


**Legend.** CI, confidence interval; MH, Mantel-Haenszel.

**Supplementary Figure 4.** Subgroup analysis for outcomes according to the percentage of female patients

Panel A: persistent/permanent AF; Panel B: cardiovascular hospitalization; Panel C: all-cause death; Panel D: adverse symptoms.


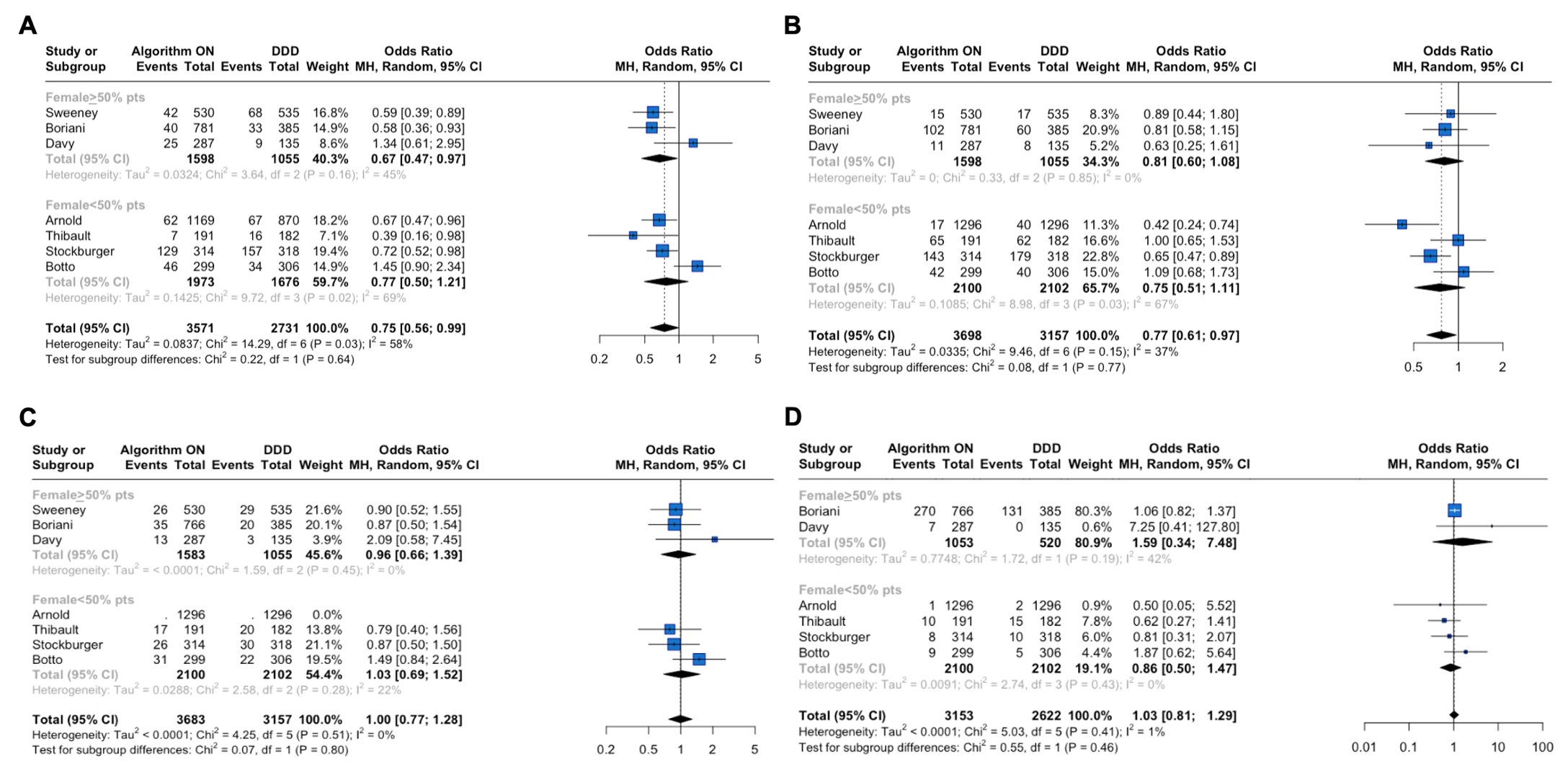


**Legend.** CI, confidence interval; MH, Mantel-Haenszel.

**Supplementary Figure 5.** Effect of algorithms on HF hospitalization and syncope.

Panel A: HF hospitalization; Panel B: syncope.


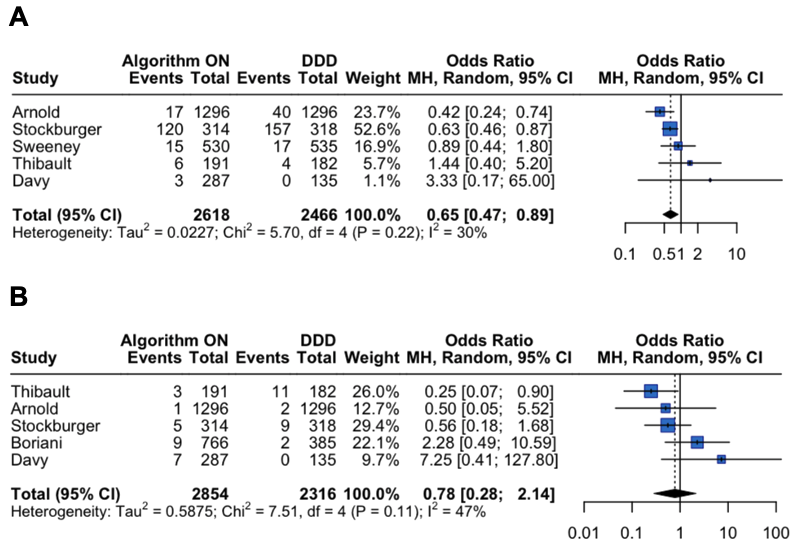


**Legend.** CI, confidence interval; HF, heart failure; MH, Mantel-Haenszel.

**Supplementary Figure 6.** Funnel plot for publication bias for outcomes.

Panel A: persistent/permanent AF; Panel B: cardiovascular hospitalization; Panel C: all-cause death; Panel D: adverse symptoms.


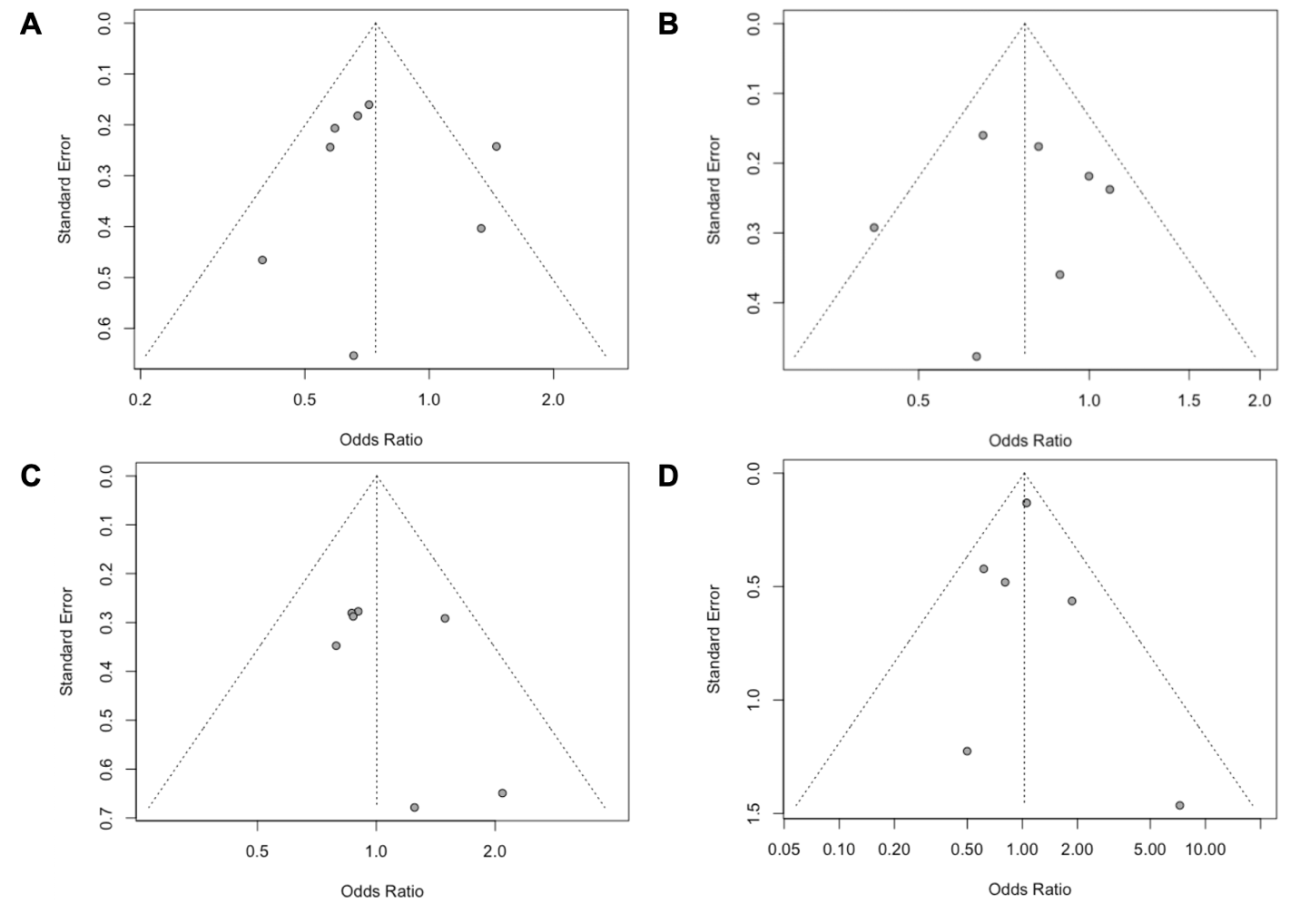


**Supplementary Figure 7.** Risk of Bias of studies included.

Panel A: Risk of bias for randomized trials; Panel B: New-castle Ottawa scale for observational study.


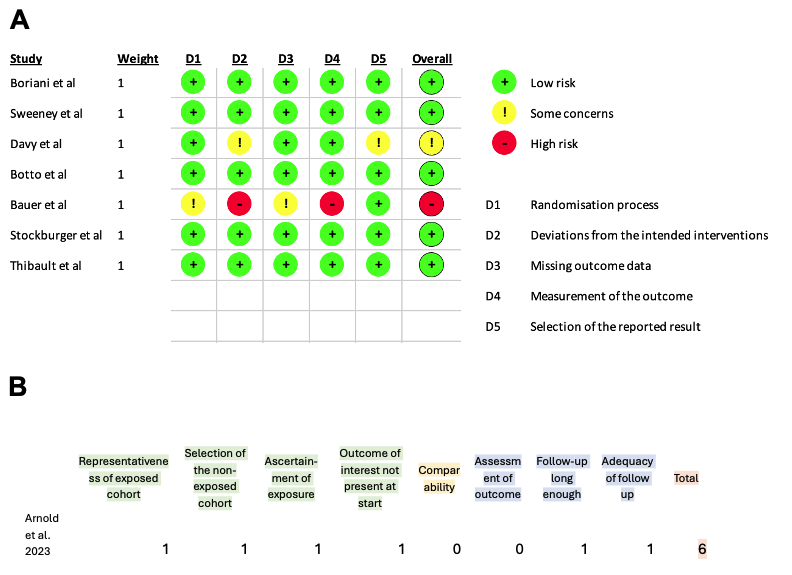


**SUPPLEMENTARY TABLES**

**Supplementary Table 1.** Characteristics of algorithms for RVPm included in the analysis

| **ALGORITHM** | **INITIAL MODE** | **SWITCH MODE TRIGGER** | **RETURN TO INITIAL PACING SETTINGS** | **ADVANTAGE/DISADVANTAGE** |
| --- | --- | --- | --- | --- |
| **VENTRICULAR INTRINSIC PREFERENCE (VIP), SJM** | DDD(R) | Intrinsic AV conduction is assessed by increasing AV delay at regular intervals for a programmed number of cycles searching for VS event, promoting intrinsic AV conduction at the longest AV detected (maximum AV delay 350 ms) | A programmed number of cycles of absent ventricular sensed events will restore initial preset. | PROS: does not allow blocked P waves  CONS: AVI extension is limited by TARP; pacing with very long AV delay may occur |
| **MANAGED VENTRICULAR PACING (MVP), MDT** | AAI(R) | Switch to DDD(R) if AV block is detected, defined as 2/4 absent ventricular events | Periodic checking for AV conduction at regular intervals (from minutes to hours); AAI mode restored when intrinsic AV conduction is found | PROS: Allows first grade AV block regardless of PR length; allows second grade AV block type 1 (Luciani-Wenckebach)  CONS: Does not allow 2:1 AV block |
| **SafeR, LivaNova** | AAI(R) | Switch to DDD(R) if any of the following is met:   - 6 consecutive PR intervals longer than the programmed long PR limit. The allowed duration of PR varies with the heart rate; the maximum allowed duration of PR intervals is programmable (AV block I criteria) - 3 out of 12 non conducted atrial events (AV block II criteria) - 2 consecutives non conducted atrial event (AV block III criteria); - ventricular pauses of 2–4 s (programmable) (AV block III criteria) |  | PROS: Allows first grade AV block regardless of PR length; allows second grade AV block type 1 (Luciani-Wenckebach); programmability  CONS: allows symptomatic 2:1 AV block. |

**Legend.** AV, atrioventricular; MDT, Medtronic; RVPm, right ventricular pacing minimization; SJM, St Jude Medical.

**Supplementary Table 2.** PRISMA checklist.

| **Section and Topic** | **Item #** | **Checklist item** | **Location where item is reported** |
| --- | --- | --- | --- |
| **TITLE** | | |  |
| Title | 1 | Identify the report as a systematic review. | Pages 1, 3, 5, 13 |
| **ABSTRACT** | | |  |
| Abstract | 2 | See the PRISMA 2020 for Abstracts checklist. | Page 3 |
| **INTRODUCTION** | | |  |
| Rationale | 3 | Describe the rationale for the review in the context of existing knowledge. | Page 5 |
| Objectives | 4 | Provide an explicit statement of the objective(s) or question(s) the review addresses. | Page 5/6 |
| **METHODS** | | |  |
| Eligibility criteria | 5 | Specify the inclusion and exclusion criteria for the review and how studies were grouped for the syntheses. | Supplementary material Page 2/3 |
| Information sources | 6 | Specify all databases, registers, websites, organisations, reference lists and other sources searched or consulted to identify studies. Specify the date when each source was last searched or consulted. | Supplementary material Page 2/3 |
| Search strategy | 7 | Present the full search strategies for all databases, registers and websites, including any filters and limits used. | Supplementary material Page 2/3 |
| Selection process | 8 | Specify the methods used to decide whether a study met the inclusion criteria of the review, including how many reviewers screened each record and each report retrieved, whether they worked independently, and if applicable, details of automation tools used in the process. | Supplementary material Page 2/3 |
| Data collection process | 9 | Specify the methods used to collect data from reports, including how many reviewers collected data from each report, whether they worked independently, any processes for obtaining or confirming data from study investigators, and if applicable, details of automation tools used in the process. | Supplementary material Page 2/3 |
| Data items | 10a | List and define all outcomes for which data were sought. Specify whether all results that were compatible with each outcome domain in each study were sought (e.g. for all measures, time points, analyses), and if not, the methods used to decide which results to collect. | Page 6-7 |
|  | 10b | List and define all other variables for which data were sought (e.g. participant and intervention characteristics, funding sources). Describe any assumptions made about any missing or unclear information. | Supplementary material Page 2/3 |
| Study risk of bias assessment | 11 | Specify the methods used to assess risk of bias in the included studies, including details of the tool(s) used, how many reviewers assessed each study and whether they worked independently, and if applicable, details of automation tools used in the process. | Page 6 |
| Effect measures | 12 | Specify for each outcome the effect measure(s) (e.g. risk ratio, mean difference) used in the synthesis or presentation of results. | Page 7 |
| Synthesis methods | 13a | Describe the processes used to decide which studies were eligible for each synthesis (e.g. tabulating the study intervention characteristics and comparing against the planned groups for each synthesis (item #5)). | Supplementary material Page 2/3 and pages 7-8 |
|  | 13b | Describe any methods required to prepare the data for presentation or synthesis, such as handling of missing summary statistics, or data conversions. | Page 8 |
|  | 13c | Describe any methods used to tabulate or visually display results of individual studies and syntheses. | Page 7-8 |
|  | 13d | Describe any methods used to synthesize results and provide a rationale for the choice(s). If meta-analysis was performed, describe the model(s), method(s) to identify the presence and extent of statistical heterogeneity, and software package(s) used. | Pages 7/8 |
|  | 13e | Describe any methods used to explore possible causes of heterogeneity among study results (e.g. subgroup analysis, meta-regression). | Page 7-8 |
|  | 13f | Describe any sensitivity analyses conducted to assess robustness of the synthesized results. | Page 7-8 |
| Reporting bias assessment | 14 | Describe any methods used to assess risk of bias due to missing results in a synthesis (arising from reporting biases). | Page 7-8 |
| Certainty assessment | 15 | Describe any methods used to assess certainty (or confidence) in the body of evidence for an outcome. | Page 7/8 |
| **RESULTS** | | |  |
| Study selection | 16a | Describe the results of the search and selection process, from the number of records identified in the search to the number of studies included in the review, ideally using a flow diagram. | Pages 8 Figure 1 |
|  | 16b | Cite studies that might appear to meet the inclusion criteria, but which were excluded, and explain why they were excluded. | Pages 9, Figure 1, |
| Study characteristics | 17 | Cite each included study and present its characteristics. | Table 1, 8-19 |
| Risk of bias in studies | 18 | Present assessments of risk of bias for each included study. | Supplemental Figure S7 |
| Results of individual studies | 19 | For all outcomes, present, for each study: (a) summary statistics for each group (where appropriate) and (b) an effect estimate and its precision (e.g. confidence/credible interval), ideally using structured tables or plots. | Pages 8-13 Figure 2, Supplemental Figures |
| Results of syntheses | 20a | For each synthesis, briefly summarise the characteristics and risk of bias among contributing studies. | Pages 8-13, Figure 7, Supplemental Figures |
|  | 20b | Present results of all statistical syntheses conducted. If meta-analysis was done, present for each the summary estimate and its precision (e.g. confidence/credible interval) and measures of statistical heterogeneity. If comparing groups, describe the direction of the effect. | Pages 8-13, Figure 2, Supplemental Figures |
|  | 20c | Present results of all investigations of possible causes of heterogeneity among study results. | Pages 8-13 |
|  | 20d | Present results of all sensitivity analyses conducted to assess the robustness of the synthesized results. | Pages 8-13 |
| Reporting biases | 21 | Present assessments of risk of bias due to missing results (arising from reporting biases) for each synthesis assessed. | Pages 8-13 |
| Certainty of evidence | 22 | Present assessments of certainty (or confidence) in the body of evidence for each outcome assessed. | Pages 8-13 |
| **DISCUSSION** | | |  |
| Discussion | 23a | Provide a general interpretation of the results in the context of other evidence. | Pages 13-18 |
|  | 23b | Discuss any limitations of the evidence included in the review. | Pages 13/18 |
|  | 23c | Discuss any limitations of the review processes used. | Pages 13/18 |
|  | 23d | Discuss implications of the results for practice, policy, and future research. | Pages 13/17 |
| **OTHER INFORMATION** | | |  |
| Registration and protocol | 24a | Provide registration information for the review, including register name and registration number, or state that the review was not registered. | n/a |
|  | 24b | Indicate where the review protocol can be accessed, or state that a protocol was not prepared. | n/a |
|  | 24c | Describe and explain any amendments to information provided at registration or in the protocol. | n/a |
| Support | 25 | Describe sources of financial or non-financial support for the review, and the role of the funders or sponsors in the review. | Page 20 |
| Competing interests | 26 | Declare any competing interests of review authors. | Page 2 |
| Availability of data, code and other materials | 27 | Report which of the following are publicly available and where they can be found: template data collection forms; data extracted from included studies; data used for all analyses; analytic code; any other materials used in the review. | n/a |

*From:*  Page MJ, McKenzie JE, Bossuyt PM, Boutron I, Hoffmann TC, Mulrow CD, et al. The PRISMA 2020 statement: an updated guideline for reporting systematic reviews. BMJ 2021;372:n71. doi: 10.1136/bmj.n71
